# *Notas Desde el Campo:* Brote de Dengue — Perú, 2023

**Published:** 2024-02-01

**Authors:** César V. Munayco, Betsabet Yadira Valderrama Rosales, Susan Yanett Mateo Lizarbe, Carmen Rosa Yon Fabian, Ricardo Peña Sánchez, César Henry Vásquez Sánchez, Maria Paquita García, Carlos Padilla-Rojas, Victor Suárez, Liliana Sánchez-González, Forrest K. Jones, Luciana Kohatsu, Laura E. Adams, Juliette Morgan, Gabriela Paz-Bailey

**Affiliations:** ^1^Centro Nacional de Epidemiología, Prevención y Control de Enfermedades, Ministerio de Salud, Lima, Perú; ^2^Ministerio de Salud, Lima, Perú; ^3^Instituto Nacional de Salud, Ministerio de Salud, Lima, Perú; ^4^División de Enfermedades Transmitidas por Vectores, Centro Nacional de Enfermedades Infecciosas Emergentes y Zoonóticas, CDC; ^5^Servicio de Inteligencia Epidemiológica, CDC; ^6^Oficina Regional para Sudamérica, CDC.

**Resumen**

**¿Qué se sabe ya sobre este tema?**

El dengue, una enfermedad viral transmitida por mosquitos, es endémico en Perú, con una cantidad anual de casos que osciló entre 4,698 y 68,290 entre los años 2017 y 2022.

**¿Qué se agrega con este informe?**

En marzo del 2023 hubo un fuerte aumento de casos de dengue en Perú: Se notificaron 222,620 casos de dengue y 381 muertes asociadas al dengue en las primeras 30 semanas del año, lo cual es 10 veces más alto que el promedio de los 5 años anteriores. En el área metropolitana de Lima se vio una carga sustancialmente más alta, en comparación con los pocos casos de transmisión local observados históricamente.

**¿Cuáles son las implicaciones para la práctica de salud pública?**

Los brotes de dengue pueden ser explosivos y sobrecargar los sistemas de atención médica, por lo cual se requiere su rápida detección, además de preparación y esfuerzos intensivos para fortalecer la capacidad de respuesta a nivel de atención médica primaria.

El dengue, una enfermedad viral transmitida por mosquitos, es endémico en Perú y la temporada de mayor transmisión suele ser entre noviembre y mayo (*1*). Los cuatro virus del dengue (DENV 1–4) han circulado en Perú, más comúnmente el DENV-1 y el DENV-2 (*2*). Históricamente, los departamentos (el primer nivel de subdivisión administrativa) del norte han notificado la mayor incidencia del dengue, mientras que, en el área metropolitana de Lima, en la costa central del Pacífico (población aproximada de 11 millones) la incidencia ha sido baja.

## Hallazgos Epidemiológicos 

En marzo del 2023, la media semanal de casos de dengue en Perú aumentó marcadamente (figura), de 2182 durante las semanas epidemiológicas 1–10 (que corresponden al periodo del 1.^o^ de enero al 11 de marzo) a 8787 durante las semanas 11–20 (del 12 de marzo al 20 de mayo). Al final de la semana 30 (29 de julio), los 222,620 casos notificados en el 2023 fueron aproximadamente 10 veces mayor que el promedio del mismo periodo durante los 5 años anteriores (21,841 casos) y 3.5 veces mayor que el número reportado durante el mismo periodo en el 2017 (64 431 casos), el año del mayor brote nacional previo de dengue. El 21 de abril se emitió una alerta epidemiológica a nivel nacional para notificar a los proveedores de atención médica acerca del riesgo de brotes de dengue. Empleados de los CDC fueron movilizados a Perú a finales de mayo para colaborar en la investigación del brote. Esta actividad fue revisada por los CDC, no se consideró investigación y se llevó a cabo de acuerdo con la legislación federal aplicable y la política de los CDC.*

**FIGURA:**
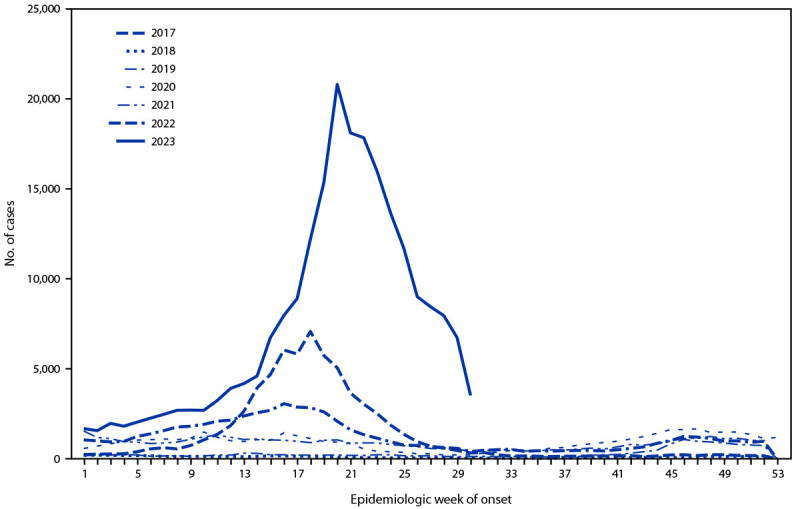
Cantidad semanal de casos de dengue notificados en todo el país,* por semana epidemiológica^†^ — Perú, Enero 1, 2017–Julio 29, 2023 * Población = 34 millones. ^†^ Las semanas epidemiológicas comienzan el domingo y terminan el sábado. Los datos del 2023 se muestran hasta la semana epidemiológica 30.

Durante el periodo del 1.^o^ de enero al 29 de julio, se reportó un total de 83 254 casos probables^†^ y 139,366 casos confirmados^§^ de dengue, lo que convirtió este brote de dengue en el brote más grande registrado en la historia de Perú. Varios de los departamentos^¶^ con las mayores cantidades de casos (ubicados en la zona costera del noroeste de Perú), incluidos Piura (67,697), Lambayeque (28,235) y La Libertad (20,289) (Tabla Suplementaria, https://stacks.cdc.gov/view/cdc/147147), también notificaron una alta incidencia durante el brote del 2017 y se vieron afectados por lluvias extremas a principios de marzo del 2023 en relación con el ciclón Yaku.** La cantidad de casos en Lima (32,009) fueron mucho más altas que aquellas notificadas en los años anteriores, incluso en los vecindarios que históricamente no han notificado casos de dengue. La mayor incidencia por edad (807 casos por cada 100,00 miembros de la población) se reportó entre las personas de 12-17 años; el 55% de los casos fueron en mujeres.

## Mortalidad 

En general, se notificaron 381 muertes relacionadas con el dengue (tasa de letalidad [CFR] = 0.17%). Más de la mitad de todas las muertes (204, o el 54%) fueron en personas de ≥60 años, en quienes también se registró la CFR más alta (0.90%), y casi una tercera parte de las muertes relacionadas con el dengue (109, o el 29%) fueron en personas de 30–59 años (CFR = 0.13%). Las personas de <30 años con dengue registraron la menor cantidad de muertes (68, o el 18%) y tuvieron la CFR más baja (0.06%).

La mayor cantidad de muertes se registró en Piura (130 muertes, CFR = 0.19 %), luego en Lambayeque (115 muertes, CFR: 0.41 %) e Ica (52, CFR = 0.32 %). Se notificaron muertes relacionadas con el dengue en 16 (64 %) de las 25 jurisdicciones.

## Pruebas de Diagnóstico 

A través de una red de 49 laboratorios de salud pública se realizaron pruebas de diagnóstico moleculares y serológicas, como pruebas de reacción en cadena de la polimerasa con transcripción inversa en tiempo real, ensayos de inmunoadsorción enzimática de antígenos de la proteína no estructural 1 y ensayos de inmunoadsorción enzimática de anticuerpos inmunoglobulina M. Estos laboratorios realizaron más de 200 000 pruebas en el 2023. Entre los 14,462 casos con el serotipo DENV disponibles en el 2023, el serotipo más común identificado fue el DENV-2 (7105, o el 49%), seguido del DENV-1 (7038, o el 49%) y el DENV-3 (319, o el 2%).^††^

## Conclusiones Preliminares y Cursos de Acción 

El Ministerio de Salud de Perú, en colaboración con las oficinas de salud regionales y colaboradores internacionales, implementó una estrategia amplia e integrada de vigilancia y respuesta, la cual incluyó más tratamientos larvicidas selectivos en aguas estancadas y fumigación con insecticida en los vecindarios afectados. En las zonas donde hubo brotes se establecieron unidades de vigilancia clínica con personal especializado y capacitado en el manejo clínico del dengue, y los hospitales implementaron carpas para el triaje de los pacientes febriles. Asimismo, hubo capacitaciones presenciales y en línea para los médicos de todo el país.

El dengue es una amenaza cada vez mayor para la salud a nivel mundial, y hay múltiples factores que contribuyen potencialmente a su creciente incidencia y expansión a nuevas áreas; por ejemplo, la rápida urbanización, el aumento en la cantidad de viajes y el cambio climático (*3*). Los brotes de dengue pueden ser explosivos y un reto para los sistemas de atención médica, por lo cual requieren el rápido reconocimiento de la transmisión, y preparación y esfuerzos intensivos para fortalecer la capacidad de respuesta a nivel de atención médica primaria. Para reducir la morbilidad y la mortalidad del dengue, es cada vez más crítico contar con intervenciones y recursos adicionales, lo que incluye vacunas y métodos de control de vectores que sean eficaces y se puedan aumentar a escala (*4*). Las agencias de salud pública pueden prepararse y responder a los brotes de dengue al evaluar y apoyar la implementación de vacunas y métodos eficaces de control de vectores, fortalecer la vigilancia del dengue y reforzar la capacitación en su manejo clínico para mejorar los resultados de los pacientes.

## Footnotes

* 45 C.F.R. parte 46, 21 C.F.R. parte 56; 42 U.S.C. Sección. 241(d); 5 U.S.C. Sección 552a; 44 U.S.C. Sección. 3501 et seq.

† Los casos probables se definen como enfermedad febril de ≤7 días de duración en una persona que reside en un área de transmisión del virus del dengue o de infestación de *Aedes aegypti*, o ha visitado una de estas áreas, dentro de los 14 días anteriores al inicio de los síntomas y presenta al menos dos de los siguientes signos y síntomas: dolor ocular o retroorbitario, mialgia, dolor de cabeza, artralgia, dolor lumbar, erupción cutánea y náuseas ovómitos. Además, los casos probables no tienen resultados de pruebas de laboratorio ni vínculo epidemiológico a otros casos de dengue.

§ Los casos confirmados tienen los mismos síntomas y criterios de exposición que los casos probables, pero han recibido al menos un resultado positivo en una o más de las siguientes pruebas: 1) aislamiento del virus en cultivo celular, 2) reacción en cadena de la polimerasa cuantitativa con transcripción inversa en tiempo real, 3) ensayo de inmunoadsorción enzimática (ELISA) de antígenos de la proteína no estructural 1, 4) detección de anticuerpos de inmunoglobulina M (IgM) del dengue en una sola muestra mediante ELISA (en áreas donde el dengue sea endémico), 5) evidencia de seroconversión de IgM en muestras emparejadas (en áreas donde no haya transmisión del dengue, y los casos deben tener una investigación epidemiológica).

¶ https://www.dge.gob.pe/sala-situacional-dengue/#grafico01

**
https://www.reuters.com/business/environment/heavy-rainfall-peru-slammed-infrastructure-losses-seen-323-mln-2023-03-22/

†† https://app.powerbi.com/view?r=eyJrIjoiOTQ0MzllOTItNWNkNC00MzE3LWJiM2QtZGUyYjU0NWFjYjUyIiwidCI6ImI0NzYxY2VlLTlkYWQtNDc3MS05ZjQ3LTVmYjc4Y2MxYjRhYSIsImMiOjR9&pageName=ReportSection73939390533a7a82da04 (Consultado el 26 de enero del 2024).
